# Transcriptomic Changes in Broiler Chicken Hypothalamus during Growth and Development

**DOI:** 10.1155/2018/6049469

**Published:** 2018-10-14

**Authors:** Katarzyna Piórkowska, Kacper Żukowski, Katarzyna Połtowicz, Joanna Nowak, Dorota Wojtysiak, Natalia Derebecka, Joanna Wesoły, Katarzyna Ropka-Molik

**Affiliations:** ^1^Department of Molecular Biology, National Research Institute of Animal Production, Balice 32-083, Poland; ^2^Department of Cattle Breeding, National Research Institute of Animal Production, Balice 32-083, Poland; ^3^Department of Poultry Breeding, National Research Institute of Animal Production, Balice 32-083, Poland; ^4^Institute of Veterinary Sciences, Department of Animal Anatomy, Agricultural University of Cracow, Kraków 30-059, Poland; ^5^Laboratory of High Throughput Technologies, Institute of Molecular Biology and Biotechnology, Faculty of Biology, Adam Mickiewicz University, 61-614 Poznań, Poland

## Abstract

The hypothalamus plays an overarching role that is reflected in the physiological processes observed in the entire organism. The hypothalamus regulates selected metabolic processes and activities of the autonomic nervous system. The avian hypothalamus due to the structural complexity is not well described and has a slightly different function than the mammalian hypothalamus that is the subject of numerous studies. The present study evaluated activities of hypothalamic genes in fast-growing chickens during development (at the 1^st^ day and 3^rd^ and 6^th^ weeks after hatching). The hypothalamic transcriptomes for 3- and 6-week-old cockerels were analysed using an RNA sequencing method in next-generation sequencing (NGS) technology. The differentially expressed gene analysis was conducted using DESeq2 software. In younger 22-day-old cockerels, 389 genes showed higher expression (fold change > 1.5) than that in 45-day-old birds. These genes played a role in several biological processes because they encoded proteins involved in integrin signalling, regulation of hormone levels, camera-type eye development, and blood vessel development. Moreover, surprisingly in the hypothalamus of 3-week-old cockerels, transcripts were identified for proteins involved in both anorexigenic (*POMC*, *NMU)* and orexigenic (*PMCH*, *ALDH1A1*, *LPL*, and *GHRH*) pathways. The RNA-seq results were confirmed by qPCR methods. In summary, the intensive growth of 3-week-old chickens was reflected in hypothalamic activities because the genes associated with the somatotropin axis and regulation of satiety centre showed increased expression.

## 1. Introduction

The hypothalamus is the part of the brain localized below the thalamus and dorsally to the anterior of the pituitary. The primary role of the hypothalamus is to link the nervous and endocrine systems [1] and, in this role, synthesizes and releases special neurohormones called releasing hormones and neurotransmitters that stimulate subsequent pituitary secretion. The hypothalamus is responsible for the regulation of thirst and hunger, sleep and circadian rhythms, body temperature, and fatigue in organisms [2, 3]. Developmental abnormalities of the hypothalamus result in disturbance during growth and sexual maturation. Moreover, such abnormalities result in physiological and neurological alterations causing obesity, autism, infertility, depression, and chronic stress [4].

During organismal development, the hypothalamus plays a significant role because the action of growth hormone (GH) is mediated; thus, body composition, fat utilization, somatic growth, physical strength, and agility are affected. Moreover, the hypothalamus controls the intermediary metabolism and secretes important hormones such as GH-releasing hormone (GHRH), somatostatin (SST), and ghrelin (GHRL) that regulate GH action. Additionally, impaired GH secretion results in growth retardation in children [5].

With the development of molecular biology tools, experimentation is possible in dimensions inaccessible ten years ago. For example, a novel high-throughput RNA sequencing (RNA-seq) method identified the *ASB2* gene as a gene candidate for pectoralis muscle tenderness in broiler chickens [6]. Moreover, Han et al. [7] using an RNA-seq method concluded that B-cell development could be affected by noncoding RNAs expressed in the bursa of Fabricius in Silky Fowl chicken strain. Han et al. [8] evaluating the hypothalamic transcriptome provided new insight into miRNA functions involved in timing the rapid development of chicken gonads. Additionally, a study conducted for the doctoral thesis of Kamineni [9] showed that heat stress significantly upregulated two genes encoding rate-limiting enzymes, lipoprotein lipase (*LPL*), and sterol regulatory element-binding transcription factor 1 (*SREBF1*).

In birds, the hypothalamic regions cannot be analysed separately for their functionality, whereas in mammals, such analysis is possible thanks to Nissl staining [10]. Therefore, bird hypothalamic areas are not well described, and the avian hypothalamus remains a curiosity for researchers and hides many mysteries. The aim of the present study was to identify genes and metabolic processes involved in growth and development in broiler chickens that are activated during ontogenesis in the hypothalamus.

## 2. Material and Methods

### 2.1. Animals

For the avian model, 24 fast-growing Ross 308 cockerels were used. The cockerels were hatched on the same day in a commercial poultry hatchery. The eggs were obtained from selected parent stock farms and delivered to the experimental farm of the National Research Institute of Animal Production located in Aleksandrowice (Poland). The chickens were fed ad libitum complete starter (days 1–21), grower (days 22–35), and finisher (days 36–45) diets containing 22, 20.5, and 20.5% completed protein (CP), respectively, and 2990, 3130, and 3130 kcal/kg ME, respectively. The mixtures were composed according to dietary requirements for meat-type chickens [11] and ensured rapid development to market size.

**Figure 1:**
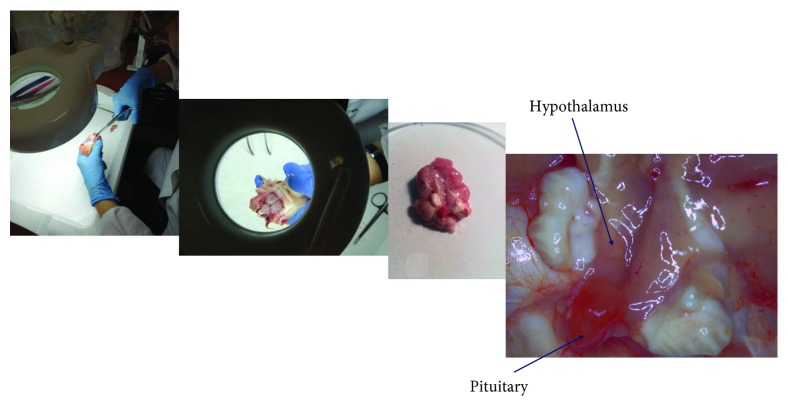
The isolation of the hypothalamus using the magnifier Benefit 130E Pro7 and minitweezers.

The chickens were kept in pens on deep litter in the same optimal, electronically controlled environmental conditions (temperature, lighting regime, and air humidity) until 1 (*n* = 8), 22 (*n* = 8) and 45 days of age (*n* = 8). The 22-day-old cockerels reached an average of 1.2 kg of body weight (BW), and the 45-day-old cockerels averaged 3.5 kg. The chickens were euthanized by decapitation. Within 20 minutes after slaughter, the hypothalamus was dissected using a magnifier 130E Pro7 (Benefit, Poland) and minitweezers (Figure 1) from each brain according to the landmarks of the optic chiasm rostrally and the mammillary bodies caudally that were described by Xu et al. [12].

Dissected hypothalamic tissues were quickly collected into tubes with RNAlater® solution (Ambion) and then were left at +4°C for 24 h before being frozen at −20°C. Carcass and growth traits were determined in accordance with the methods described by Połtowicz et al. [13]. All procedures followed were in accordance with the ethical standards of the responsible committee on animal experimentation (institutional and national).

### 2.2. cDNA Library Construction and NGS Sequencing

RNA was isolated with PureLink™ RNA Mini Kit (Ambion) according to manufacturer's recommendations. Hypothalamus samples were homogenized with beads using a Bullet Blender24 homogenizer (Next Advance). RNA was purified by an Agencourt® RNAClean™ XP (Beckman Coulter, Inc.). Quantitative and qualitative evaluation of RNA was performed using a TapeStation 2200 (Agilent). The RIN was in the range 7.3–8.4. The cDNA libraries were prepared for two age groups: 22- and 45-day-old chickens, with a TruSeq RNA Sample Preparation Kit v2 (Illumina) according to the protocol. The libraries were indexed using individual adaptors in the order shown in Table S1 and normalized to 10 nM after which the libraries were pooled. Flow-cell clustering was performed using a TruSeq SR Cluster Kit v3-cBot HS. The sequencing was conducted on a HiScanSQ System (Illumina) in single 101-bp cycles using TruSeq Kit v3-HS chemistry according to Ropka-Molik et al. [14].

### 2.3. Alignment

The FastQC program was used for read quality control, and the Flexbar program was used to remove adapters and poly-A sequences. Reads shorter than 32 and quality scores lower than 20 were removed from the dataset. Basecalls were performed using CASAVA version 1.8.2. The cleaned reads were aligned to Gallus_gallus-5.0 (GCA_000002315.3) with a reference annotation containing 18,346 genes listed in the Ensembl database. Alignment and estimation of gene expression level were made using the RSEM [15]. The alignment and differentially expressed gene (DEG) statistics were performed using SAMStat and RNA-SeQC. The sequence data are submitted to the Gene Expression Omnibus (GSE104255).

### 2.4. Differentially Expressed Gene Analysis and Validation

DEG analysis was conducted using DESeq2 [16]. The corrected *P* values (≤0.05) were presented as the false discovery rate (FDR). The genes that showed fold change (FC) ≥ 1.5 between chickens of different ages were included for further analysis. Gene functional analysis was performed with STRING v.10.5 and PANTHER. The “enriched by genes biological processes,” “molecular functions,” and pathways were estimated, taking into account the included FDR < 0.05 and Bonferroni correction (*P* < 0.05).

Fifteen transcripts were validated by qPCR using *RPL4* as the endogenous control [17]. The primers, probes, and assays that were used for validation are presented in Table S2 (goo.gl/tC9GTZ). The comparison of qPCR and RNA-seq results was performed by Pearson's correlation. The significant differences between gene expression levels were determined by ANOVA (Duncan's post hoc test; SAS Enterprise v. 7.1). Expression analysis of the genes of interest was conducted by the qPCR method and included all three age groups of chicken.

## 3. Results

### 3.1. Cockerel Characteristics

Several growth and carcass traits of the cockerels were measured during growth and in the dissection. After the sixth week, cockerels were nearly 3-fold heavier than the 3-week-old birds, although the growth rate of birds gradually decreased with age (Table 1).

The 3- and 6-week-old cockerels presented similar fat content and did not differ regarding the percentage of leg muscles. The share of breast muscle between the 1^st^ and the 45^th^ day after hatching increased more than 16-fold. By contrast, the leg muscle share increased only 1.7-fold during the same period.

### 3.2. RNA Sequencing Statistics

The average total number of raw reads per sample was 23,947,897 ± 1,543,653. After the removal of adapters, trimming and filtering feature 90.5% of raw reads remained that were mapped to the chicken reference genome (Galgal 5.0) of which 69.2% and 8.5% matched annotated exonic and intronic regions, respectively (see Table S1, goo.gl/tC9GTZ).

### 3.3. Differentially Expressed Gene Analysis

**Figure 2:**
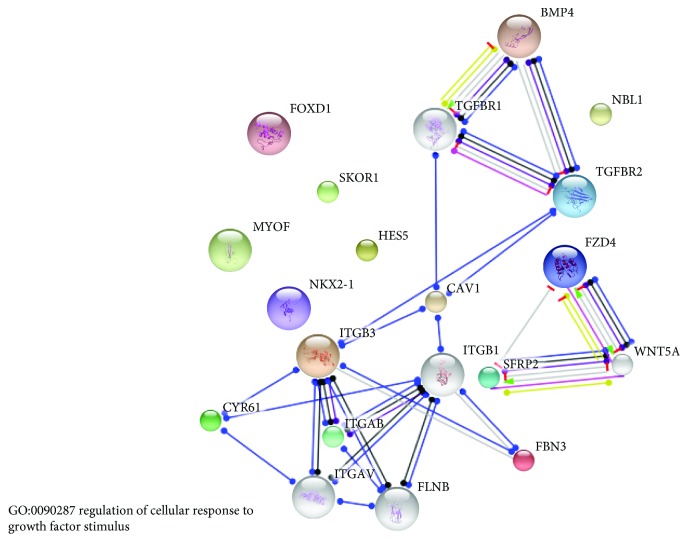
Regulation of cellular response to growth factor stimulus (GO:0090287) biological process enriched by genes that showed higher expression level in the hypothalamus of 3-week-old than 6-week-old cockerels. The grey genes are the background (connections) for upregulated genes.

Age-dependent gene expression analysis of chicken hypothalamus was performed using the DESeq2 method. The comparison of 22 and 45-day-old cockerel transcriptomes indicated 389 genes with elevated expression in younger birds (FC ≥ 1.5, adjusted *P* value ≤ 0.05). These genes encoded proteins primarily involved in integrin signalling pathways (*ACTA2*, *PIK3C2G*, *COL4A6*, *CAV1*, *LAMA1*, *FN1*, and *ITGA8*), regulation of hormone levels (*NR5A1*, *TIPARP*, *ACE*, *CRYM*, *CGA*, *ALDH1A1*, *TBX3*, *POMC*, *GHRH*, and *ALDH6*), camera-type eye development (*TGFBR2*, *BMP4*, *FABP7*, *STRA6*, *SIX6*, and *WNT6*), blood vessel development (Figure S1, goo.gl/tC9GTZ), and regulation of cellular response to growth factor stimulus (see Figure 2).

**Figure 3:**
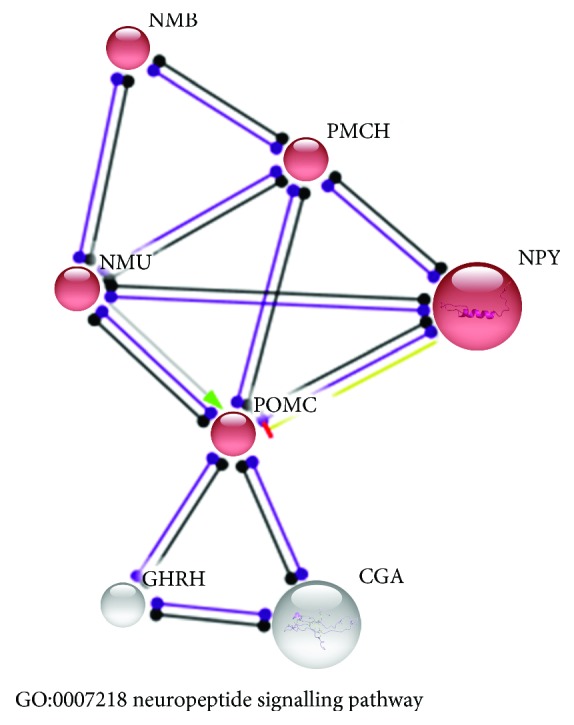
Neuropeptide signalling pathway (GO:0007218) enriched by genes that showed higher expression level in the hypothalamus of 3-week-old than 6-week-old cockerels. All presented genes were upregulated in 3-week-old chickens.

In 3-week-old cockerels, three genes (*LECT2*, *CGA*, and *ALDH6*) showed over tenfold higher expression than that in 6-week-old birds. The CGA protein interacts with POMC and GHRH of which the transcripts were also overexpressed in these 3-week-old birds (see Figure 3).

**Figure 4:**
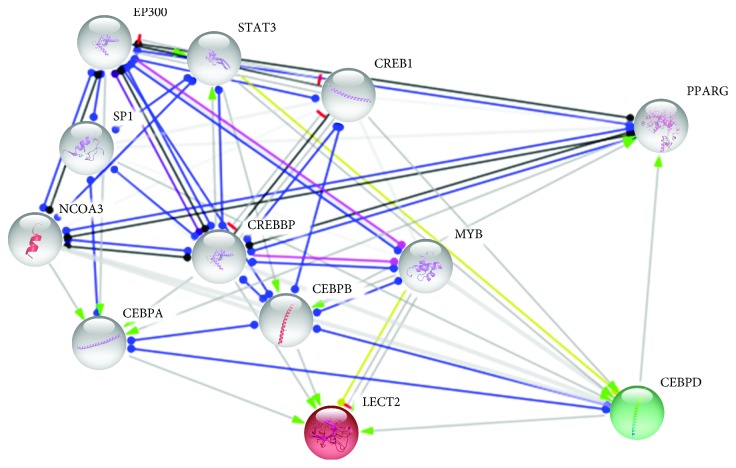
The connections of leukocyte cell-derived chemotaxin 2 (LECT2). Colored genes were upregulated in 3-week-old cockerels and grey genes are the background.

CCAAT Enhancer-Binding Protein Delta (CEBPD) induces *LECT2* expression (see Figure 4), and the *CEBPD* gene was upregulated in 3-week-old cockerels, so this type of regulation could occur in the bird hypothalamus. Moreover, in 3-week-old cockerels, the overexpression of 24 long noncoding RNAs was observed.

In the hypothalamus of 45-day-old cockerels, 246 overexpressed genes were related to neurogenesis (*MAPK9*, *HNC1*, *APOD*, *TCF7*, *POU3F1*, *GBX1*, and *KNCNQ2*), neurological signal transmission (*RIMS4*, *SV2C*, *CNTNAP2*, *GRM5*, *MAGI2*, *SV2B*, *CYSLTR1*, *SLC5A7*, and *CHRNB3*), and neurotransmitter secretion (*SYTL5*, *WNT7*, *SLC1A*, *SYN3*, *GAD1*, *SYNJ1*, *SYT9*, *SCL5A7*, *OTOF*, and *RAB3C*) (see Figure S2, goo.gl/tC9GTZ). The significant highest FC in the older cockerels was for *MAPK10* genes (see Figure S3). Mitogen-activated protein kinase 10 (MAPK10) participates in important signalling pathways such as MAPK, Toll-like receptor, TNF, and GnRH signalling pathways. All overexpressed genes (significant and with FC > 1.3) of both groups are available at the link goo.gl/RL6Y7f.

### 3.4. qPCR Analysis

**Figure 5:**
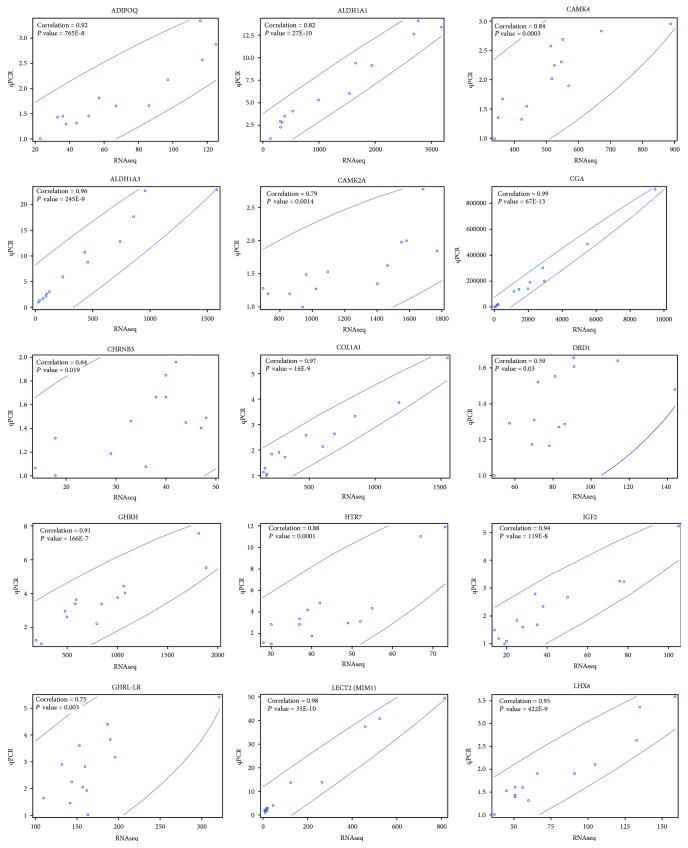
The validation of RNA-seq results by qPCR and Pearson correlation. The charts were generated with SAS Enterprise v. 7.1.

**Figure 6:**
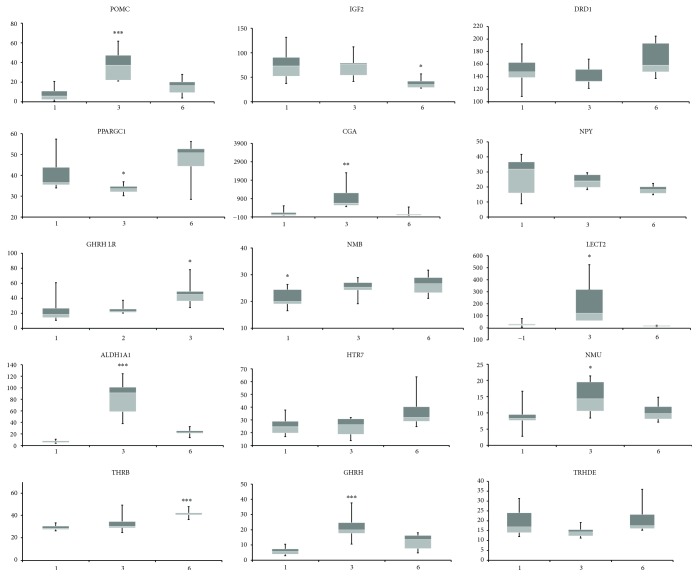
The relative expression patterns of the most notable hypothalamic transcripts dependent on broiler age (^∗^ *P* value ≤ 0.05; ^∗∗^ *P* value ≤ 0.01; ^∗∗∗^ *P* value ≤ 0.001). The genes of interest were normalized to the *RPL4* gene.

Fifteen DEGs were validated by the qPCR method and then by Pearson's correlation. The results are presented in Figure 5. The average *R* coefficient was 0.82 (*P* value = 61*E* − 50). The lowest Pearson *R* coefficient was obtained for the *DRD1* gene (*R* = 59, *P* value = 0.03) that could be associated with unrecognized isoforms of the *DRD1* gene. The most notable gene expression patterns of the 15 genes among the 3 age groups are presented in Figure 6.

## 4. Discussion

In vertebrates, the hypothalamus is a brain structure accountable for numerous behavioural, autonomic, and endocrine responses that are involved in regulating metabolism, homeostasis, and reproduction. The avian hypothalamus is not well recognized because of the lack of clear boundaries delineated by Nissl staining [10]. Moreover, the hypothalamus plays a slightly different role in birds than in mammals, for example, the role in circadian rhythms. The present report describes the hypothalamic activities by RNA-seq and qPCR during ontogenesis in chickens (Ross 308 broilers) characterized by a high growth rate.

The Ross 308 broilers are a fast-growing commercial chicken line with growth rates that are 10–15% higher than those of other commercial lines between the ages of 30 and 40 days. The slaughter weight of chickens of the Ross 308 line is obtained between the 35^th^ and 42^nd^ day from hatching, because their abdominal fat content increases significantly after this period, which is undesirable. The producer of this commercial chicken line informs in its instructions [18] that 22-day-old Ross 308 cockerels reach an average of 1 kg of body weight (BW), with 12% daily feed intake calculated for BW and 8% daily gains (DG) of BW. In turn, the BW, feed intake, and DG values of 45-day-old birds oscillate at approximately 3.2 kg, 7%, and 3.6%, respectively. The share of leg muscles elevates by a few percentages between the 3^rd^ and 6^th^ week of age, and the share of *pectoralis* muscles increases 2-fold during this period [19].

Numerous notable observations were produced in the comparison of hypothalamic activity at the transcriptome level in broiler chickens between the 3^rd^ and 6^th^ week from hatching. The Ross 308 broiler chickens according to the producer's instructions show an increase in feed intake and daily gain calculated per BW. Our study also confirmed a significant increase in growth rate in the younger 3-week-old chickens that was associated with an increase in ADG calculated per BW and could be a consequence of increased feed intake. However, in the present study, daily feed intake was not recorded for individuals. Nevertheless, this intensive growth rate was reflected in the hypothalamic activity, because numerous genes associated with hormone status regulation were overexpressed (*CGA*, *ALDH1A1*, *TBX3*, *IGF2*, *POMC*, *GHRH*, and *ALDH6*). One of the most important *GHRH* encodes somatocrinin, a key regulator of somatotropin, a growth hormone. In the 3-week-old broilers, the level of *GHRH* transcript was 2-fold higher than that in the 45-day-old broilers and over 3-fold higher than that in the 1-day-old cockerels. In chickens, the somatotropic axis is regulated by GHRH, thyrotropin-releasing hormone (TRH), and somatostatin by the secretion of the growth hormone (GH) [20]. The GH acts directly on development and metabolism and indirectly binds to GHR on liver membranes to activate insulin-like growth factor-I (IGF1) that subsequently stimulates the differentiation and proliferation of muscle and bone cells [21]. McGuinness and Cogburn [22] observed that circulating hepatic IGF1 reaches a plateau between 3 and 7 weeks of age in chickens. In the present study, insulin-like growth factor II showed a high expression in the hypothalamus of 1-day-old and 22-day-old chickens and then decreased to reach a level 2-fold lower in 45-day-old cockerels. The role of IGF2 in chicken development is not clear; it is not known to be under GH axis control, but Armstrong et al. [23] showed that IGF2 has a high affinity for the IGF1 receptor. Therefore, IGF2 could regulate endocrine and paracrine effects of growth in chickens. Moreover, in 22-day-old cockerels, increases in the expression of *IGFBP4* and *IGFBP7* were observed. These genes encode proteins that modulate IGF action by preventing insulin-like effects, but the exact action of IGFBPs in chicken growth and development remains to be resolved [24]. The DEG analysis also showed that another important axis associated with growth and development, the thyroid axis, was modulated during chicken ontogenesis. The thyrotropin-releasing hormone (TRH) expressed in the hypothalamus increases both plasma T3 and T4 in growing chickens by stimulating pituitary TSH [25]. The thyroid hormone (TH) is essential for normal growth. However, in adult chickens, TRH no longer has a clear thyrotropic activity, whereas its somatotropic activity depends on the feeding status of the birds [20]. Both somatotropic and thyroid axes are related because GH administration increases the circulation of T3 by suppressing its degradation. In the present study, the highest peak of *CGA* gene expression was observed in 3-week-old chickens, which was 11-fold higher than that in 6-week-old birds. The *CGA* gene encodes a glycoprotein hormone that is required for the normal biological activity of TSH [26]. Therefore, the CGA likely increases the circulating levels of the T4 hormone. Moreover, RNA-seq and qPCR results showed that two other genes related to TRH were also modulated with age: *TRHDE* and *THRB*. The *TRHDE* gene encodes an extracellular protein that specifically cleaves and inactivates the TRH [27] and showed the lowest expression level in 3-week-old chickens. According to producer instructions [18], 22-day-old cockerels double their body weight in comparison with that of 2-week-old cockerels. However, between the 38^th^ and 45^th^ day of age, they gain only 30% of BW. This finding suggests that TRH is most necessary during intensive growth since we found the factor to cleave the TRH in the hypothalamus of older chickens. However, the differential expression of TRH was not found in the present study, which indicates the action of another translational or posttranslational regulatory mechanism. In turn, THRB is one of the several thyroid hormone receptors, and the transcript level increased with age, showing the lowest expression level in the youngest 1-day-old cockerels and gradually increasing to reach 30% higher expression in 6-week-old cockerels. The role of THRB in the mediation of biological activities of THs may be gleaned from evidence showing that mutations in the human *THRB* result in generalized thyroid hormone resistance (GTHR) and the elevation of circulating TH levels [28]. Moreover, Hameed et al. [29] indicated the highly important role of hypothalamic THRB in controlling energy homeostasis in mice, with knockdown mice presenting severe obesity due to hyperphagia and reduced energy expenditure. Thus, the elevated level of *THRB* expression in chicken hypothalamus could play a similar role in preventing excessive fat deposition in older birds, because after 42 days, the percentage of abdominal fat significantly increases in broiler chickens.

Although the cockerels in the present study were fed ad libitum, the 3-week-old chickens that received a feed mixture providing 140 kcal/kg less showed higher daily gain and growth than the 6-week-old chickens. This finding could be associated with the feeding status of younger broilers since increased expression of numerous candidate genes involved in the regulation of appetites and energy balance was observed. However, the activity of hypothalamic proteins involved in the regulation of the satiety centre was unusual, because in 3-week-old cockerels, increase in transcript abundance of genes encoding proteins involved in both anorexigenic and orexigenic pathways was observed. These pathways should exclude one another because they respond to the conflicting signals of satiety and hunger. One of the most notable is the *POMC* gene that encodes a proopiomelanocortin, a precursor of anorexigenic neuropeptides (ACTH) and *α*-melanocyte-stimulating hormone (*α*MSH). *POMC* is upregulated during satiety signalling to prevent obesity [30]. Another gene, *NMU* also showed the highest expression level in this age group. *NMU* encodes neuromedin U that in mammals induces locomotor activity, grooming, and face washing behaviour [31], as well as wing flapping behaviour in layer chicks [32]. NMU also modulates the Fos-like immunoreactivity in oxytocin- and/or vasopressin-containing neurons in the hypothalamus in mammals [33]. In chickens, NMU suppresses food intake by inducing the anorexigenic pathway downstream mediated by corticotropin-releasing factor (CRF) [34], which is a candidate signal molecule for regulating appetite and energy in poultry [35]. Similarly, PPARGC1*α* with significantly reduced gene expression in the hypothalamus of 3-week-old cockerels is associated with the stimulation of food intake and the inflammatory response [36, 37]. Ma et al. [38] confirmed the previous study by testing PGC-1*α* null mice and showing that deficiency of PPARGC1*α* impairs the expression of *NPY* and *AgRP* genes, which induce food intake. Furthermore, they also reported that CAMK2*α* is involved in governing energy expenditure. In the present study, CAMK2*α* was also regulated during ontogenesis and showed significantly reduced gene expression in the hypothalamus of 3-week-old chickens. Previous reports suggest that both *CAMK2a* and *PPARGC1* are involved in the regulation of NPY and AgRP. However, in the present study, no significant differences in hypothalamic *NPY* and *AgRP* expression were observed or qPCR analysis did not confirm such findings. Nevertheless, other genes (*PMCH*, *ALDH1A1*, *LPL*, and *GHRH*) that indirectly play a role in the induction of feed intake showed increased expression levels in 22-day-old cockerels. Pro-melanin-concentrating hormone (PMCH) is a precursor of MCH, which stimulates appetite and plays a critical role in energy balance. MCH-null mice are hypophagic and lean, whereas the overexpression of MCH in the lateral hypothalamus leads to obesity and insulin resistance [39]. Aldehyde dehydrogenase 1 family member A1 (*ALDH1A1*) decreases the extracellular level of dopamine, which is an anorexigenic molecule [35]. In the present study, the *ALDH1A1* expression was nearly 5-fold higher in 3-week-old cockerels than in 6-week-old birds. Moreover, the expression level of the *DRD1* gene that encodes receptor 1 for dopamine is reduced in 3-week-old cockerels (although qPCR failed to confirm this finding). Lipoprotein lipase (LPL) regulates the metabolism of TG-rich glycoproteins and cellular uptake of PUFA signalling the hypothalamus in regulating orexigenic neuropeptides (AGRP/NPY) [40]. At the end, the *NTSR1* receptor for neurotensin involved in anorexigenic pathways [35] showed a 2-fold reduction in gene expression in the hypothalamus of the 3-week-old chickens; however, this result was not validated by qPCR.

On the other hand, in 3-week-old chickens, *LECT2* expression was over 22-fold higher than that in older chickens. In humans, the circulating LECT2 is correlated with the severity of both obesity and insulin resistance. The starvation-sensing adenosine monophosphate-activated protein kinase in H4IIEC hepatocytes negatively regulates the LECT2 expression [41]. The high expression of hypothalamic *LECT2* could also be involved in the regulation of appetite and energy, which should be considered in the future investigations.

In older 6-week-old chickens, the activated GO biological processes and enriched pathways were associated primarily with brain structure development. The gene that showed the highest age-dependent change in expression level was *MAPK10*, which encodes a protein that participates in important signalling pathways such as MAPK, Toll-like receptor, TNF, and GnRH signalling pathways and that is involved in a wide variety of cellular processes such as proliferation, differentiation, transcription regulation, and development [42]. Moreover, this kinase plays a role in the regulation of the signalling pathway during neuronal apoptosis. MAPK10 activity is inhibited by MDS1 and EVI1 complex locus (MECOM) and nuclear factor of activated T-cells 1 (NFATC1), which were also overexpressed in the 45-day-old chickens (see Figure S1).

## 5. Conclusions

In summary, the feed intake status of 3-week-old chickens was not obvious. Although they were fed ad libitum, their satiety centre did not provide an unambiguous signal of satiety, because many genes involved in the induction of feed intake also showed increased expression levels in the hypothalamus of this age group. However, this situation is also observed in rat foetal brain development in mothers fed a high-fat diet. Both the POMC and NPY mRNA expression increased, although only the anorexigenic pathways should have been activated. In the present study, the RNA-seq method also identified increased NPY expression (1.47-fold change) in the 3-week-old broilers; however, qPCR did not confirm this observation. The activation of genes associated with both anorexigenic and orexigenic pathways in 3-week-old broilers could be a consequence of the intensive growth rate at this age, which could be associated in turn with intensive feed intake; therefore, their anorexigenic pathways were activated to avoid excessive fat deposition. Simultaneously, they also grow intensively, which requires high expenditure of energy; therefore, they induced indirect orexigenic factors. However, further investigation of this focus is required.

## Figures and Tables

**Table 1:** Traits of investigated cockerels estimated after slaughtering (mean ± SD).

Trait	Group of Ross 308 cockerels		
	0-week-old (1-day-old) *n* = 8	3-week-old (22-day-old) *n* = 8	6-week-old (45-day-old) *n* = 8
Body weight (g)	43 ± 5.3^A^	1213 ± 104.7^B^	3532 ± 386.9^C^
Growth rate (%)		Days 0–22, 185.45%	Days 23–45, 97.3%
Average daily gain (g)		21 days, 83 ± 7.93^A^	42 days, 114.3 ± 18.1^B^
Abdominal fat (%)	0.05 ± 0.04^A^	0.73 ± 0.28^B^	0.90 ± 0.23^B^
Breast muscles (%)	1.53 ± 0.25^A^	17.93 ± 1.17^B^	24.70 ± 1.87^C^
Leg muscles (%)	9.90 ± 0.82^A^	5.53 ± 0.72^B^	16.50 ± 1.30^B^

^A,B,C^Values in rows with different letters show significant results (*P* ≤ 0.01). Growth rate (%) was calculated using the following formula: growth rate = (BW_f_–BW_i_/(BW_i_ + BW_f_) × 0.5) × 100, with BW_i_ as the initial body weight of the rearing period (g) and BW_f_ as the final body weight of the rearing period (g). Percentage of breast muscles, leg muscles, and abdominal fat of carcass was calculated in relation to the weight of the chilled carcass with giblets. ADG was measured as (BW on 21^st^ day − BW on 14^th^ day)/7 and (BW on 42^nd^ day − BW on 35^th^ day)/7.

## Data Availability

The datasets generated during and/or analysed during the current study are available from the corresponding author on request.

## Supplementary Materials

Supplementary Materials Table S1: overall statistics and read annotations obtained for each analysed cockerel sample describing raw and processed data. Table S2: genes used in validation during qPCR analysis and information on primers, probes, and assays purchased from Applied Biosystems. Figure S1: blood vessel development biological process enriched by genes that showed increases in expression level in the hypothalamus of 3-week-old cockerels. The grey genes are the background (connections) for upregulated genes. Figure S2: reactome pathways and GO biological processes generated by PANTHER and enriched by genes that showed increased expression levels in 6-week-old cockerels. Figure S3: the connections of mitogen-activated protein kinase 10 (MAPK10). Colored genes were upregulated in 6-week-old cockerels, and grey genes are the background.
