# Supplementary material for: Transcriptomic Changes in Broiler Chicken Hypothalamus during Growth and Development
Source: Int J Genomics. 2018 Oct 14;2018:6049469. doi: 10.1155/2018/6049469 (PMC6204183; doi:10.1155/2018/6049469)
Supplement: Supplementary Materials — Table S1: overall statistics and read annotations obtained for each analysed cockerel sample describing raw and processed data. Table S2: genes used in validation during qPCR analysis and information on primers, probes, and assays purchased from Applied Biosystems. Figure S1: blood vessel development biological process enriched by genes that showed increases in expression level in the hypothalamus of 3-week-old cockerels. The grey genes are the background (connections) for upregulated genes. Figure S2: reactome pathways and GO biological processes generated by PANTHER and enriched by genes that showed increased expression levels in 6-week-old cockerels. Figure S3: the connections of mitogen-activated protein kinase 10 (MAPK10). Colored genes were upregulated in 6-week-old cockerels, and grey genes are the background. [file 6049469.f1.pdf]

Table S1: Overall statistics and read annotations obtained for each analyzed cockerel samples 378 describing raw and processed data

Sample	ROSS308_3_1	ROSS308_3_2	ROSS308_3_3	ROSS308_3_4	ROSS308_3_5	ROSS308_3_6	ROSS308_3_7	ROSS308_3_8
Indexes/ pooling lane1;3;5;7	AR004	AR005	AR006	AR007	AR008	AR011	AR012	AR013
Total purity filtered reads sequenced	22 368 454	23 487 271	21 195 496	21 760 511	24 100 292	23 697 330	25 871 916	25 222 717
Alternative alignments	1 235 584	1 439 409	1 307 849	1 390 977	1 481 718	1 428 338	1 559 170	1 510 397
Exonic Rate	0.70	0.69	0.68	0.70	0.69	0.69	0.69	0.69
Intronic Rate	0.090	0.088	0.086	0.078	0.080	0.082	0.085	0.087
Transcripts detected	28 206	28 317	28 017	27 898	27 968	28 153	28 215	28 060
Genes detected	10 338	10 349	10 289	10 283	10 299	10 309	10 342	10 296
Base Mismatch Rate	0.005	0.004	0.005	0.004	0.004	0.004	0.004	0.004
**Alignment**								
Number of input reads	23 257 119	24 326 292	22 110 816	22 708 636	25027122	24532702	26819747	26169402
% of uniquely mapped reads	90.82	90.48	89.84	89.71	90.27	90.73	90.48	90.42
% of reads mapped to multiple loci	4.84	5.56	5.49	5.60	5.51	5.36	5.47	5.44
Sample	ROSS308_6_1	ROSS308_6_2	ROSS308_6_3	ROSS308_6_4	ROSS308_6_5	ROSS308_6_6	ROSS308_6_7	ROSS308_6_8
Indexes/pooling lane1;3;5;7	AR014	AR015	AR016	AR018	AR019	AR020	AR023	AR027
Total purity filtered reads sequenced	23 481 476	21 886 178	23 142 988	21 459 556	23 441 751	20 409 838	22 747 714	24 845 405
Alternative alignments	1 329 734	1 265 177	1 362 205	1 276 773	1 389 046	1 189 051	1 336 307	1 419 671
Exonic Rate	0.70	0.70	0.70	0.69	0.69	0.69	0.69	0.69
Intronic Rate	0.084	0.084	0.086	0.086	0.084	0.084	0.086	0.086
Transcripts detected	28 288	27 982	28 226	27 911	28 130	27 727	27 975	28 046
Genes detected	10 325	10 264	10 332	10 236	10 307	10 223	10 283	10 298
Base Mismatch Rate	0.004	0.004	0.005	0.005	0.004	0.004	0.004	0.004
**Alignment**								
Number of input reads	24 324 918	22 668 099	24 038 010	22 273 733	24329666	21233340	23586068	25760675
% of uniquely mapped reads	90.93	90.80	90.49	90.51	90.49	90.37	90.70	90.69
% of reads mapped to multiple loci	5.07	5.22	5.24	5.30	5.35	5.24	5.24	5.24

Table S2: Genes used in validation during qPCR analysis and the information about primers, probes, and assays purchased in Applied Biosystem

Gene		Primers and Probe<br>or<br>Taqman Gene Expression Assay ID	Amplicon length [bp]	Label	Reference sequences accesion numbers	PCR efficiency %	Exon Boundary
ADIPOQ	Adiponectin, C1Q And Collagen Domain Containing	Gg03311243_m1	62	VIC	BX931303.1	100	5-6
ALDH1A1	Aldehyde dehydrogenase 1 family, member A1	Gg03313288_m1	146	VIC	NM_204577.4	94	6-7
CAMK4	Calcium/calmodulin-dependent protein kinase type IV	Gg03357449_m1	73	FAM	NM_001034813.1	105	5-6
ALDH1A3	Aldehyde Dehydrogenase 1 Family Member A3	Gg03362468_m1	146	FAM	NM_204577.4	101	6-7
RPL4	Ribosomal Protein L4 **housekeeping gene**	Gg03370187_m1	75	VIC	NM_001007479.1	90	2-3
CAMK2A	Calcium/calmodulin-dependent protein kinase type II alpha chain	Gg03346980_g1	73	VIC	NM_001004375.1	99	8-9
CGA	Thyroid-Stimulating Hormone Alpha Chain	F-AGGGAGAAAGATCATGGATTGC<br>R- GCAGAAATACAGACAAAATGGTCAA<br>PROBE - ACAGGAAGTATGCAGCTGT	71	NED	ENSSSCG00000025434	98	1-2
CHRNB3	Cholinergic receptor nicotinic beta 3 subunit	Gg03338737_m1	79	FAM	NM_204812.1	97	3-4
ENSGALG00000041555	*similar to COL1A1* Collagen type I alpha 1 chain	F-TCCAGGGAGCCAACGAGAT<br>R-GTGACTCGTGCAGCCATCCT<br>PROBE - ATCAGGGCCGAAGGA	87	NED	ENSGALG00000041555	92	1-2
DRD1	Dopamine receptor D1	Gg03812156_s1	71	FAM	NM_001144848.1	101	1-1
GHRH	Growth hormone-releasing hormone	Gg03359589_m1	71	FAM	NM_001040464.1	100	3-4
HTR7	5-hydroxytryptamine receptor 7	F-TCCATCATGACACTGTGCATCA<br>R-CGTTTTGTCTCACGGGATACG<br>PROBE - CGTGGACAGGTATCTG	82	NED	ENSSSCG00000010459	93	1-2

IGF2	Insulin like growth factor 2	Gg03359160_m1	72	VIC	NM_001030342.1	100	1-2
ENSGALG00000005242	similar to GHRH-LR	F-GGTGGTGAAAGAAGAGGCACTCT<br>R-CGCATCCTTTAAGATCAGGTGAA<br>PROBE - TTAGAAAGGAACGAATCTG	68	NED	ENSGALG00000005242	97	3-4
LECT2	leukocyte cell-derived chemotaxin 2<br>2	Gg03340174_m1	73	FAM	NM_205478.1	92	5-6
LHX6	LIM homeobox 6	Gg03319857_m1	73	FAM	DQ082894.1	98	-
PPARGC1A	PPARG coactivator 1 alpha	Gg03346752_m1	70	FAM	NM_001006457.1	91	10 - 11
NMU	Neuromedin U	Gg03318848_m1	78	FAM	NM_001277921.1	93	2-3
POMC	Proopiomelanocortin	Gg03352057_m1	134	FAM	NM_001031098.1	93	1-2
NMB	Neuromedin B	Gg03360412_m1	53	FAM	NM_001079476.2	89	1-2
TRHDE	Thyrotropin-releasing hormone-degrading ectoenzyme	TGCCCTAAATATTACAAGAAGACTCA<br>TCCTTTGCTCCACAAAAACA	153	Evagreen	ENSGALT00000016581.5	93	3-4

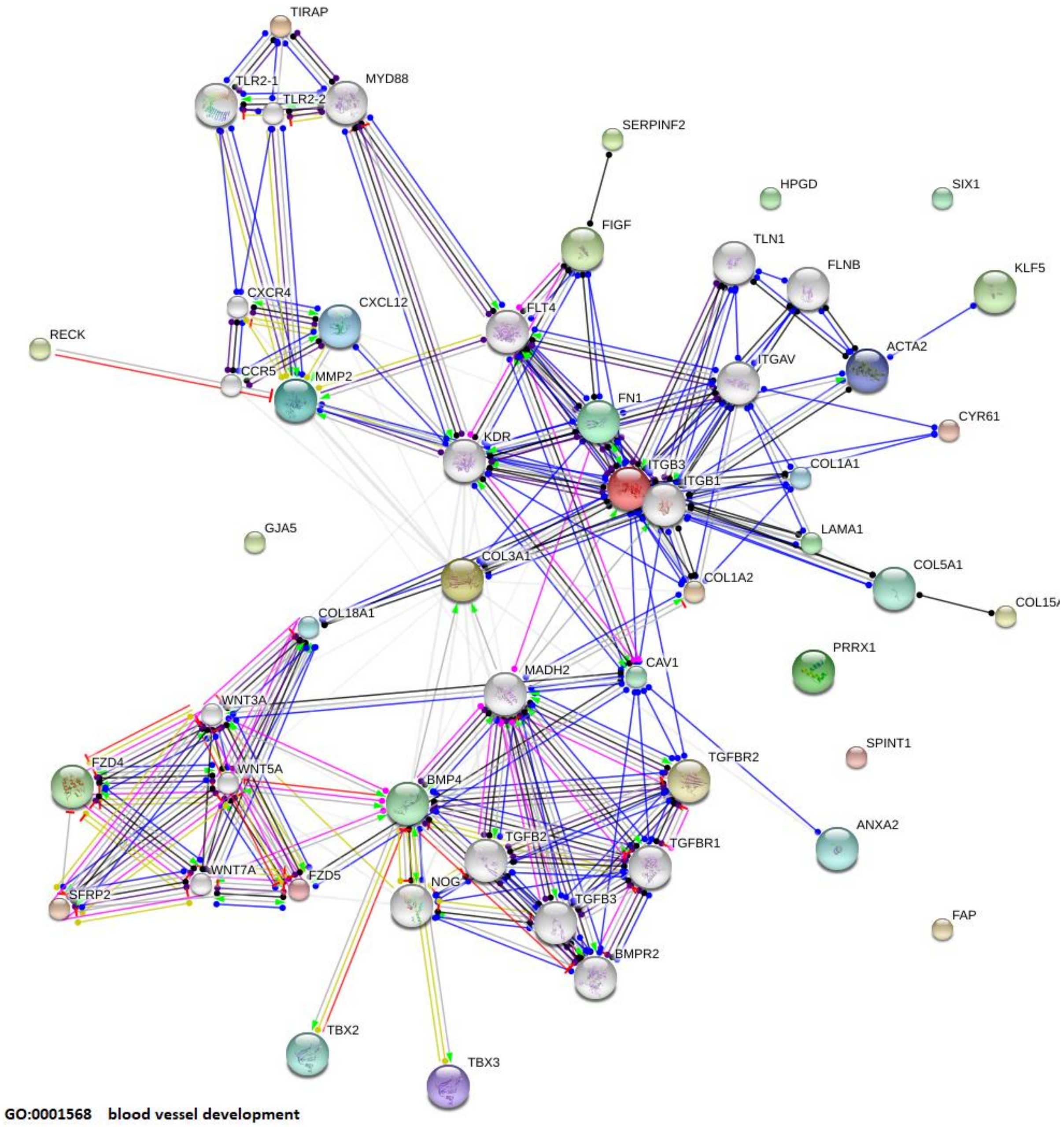

Figure S1: Blood vessel development biological process enriched by genes showed higher expression level in the hypothalamus of 3-week-old cockerels. The gray genes are the background (connections) for upregulated genes.

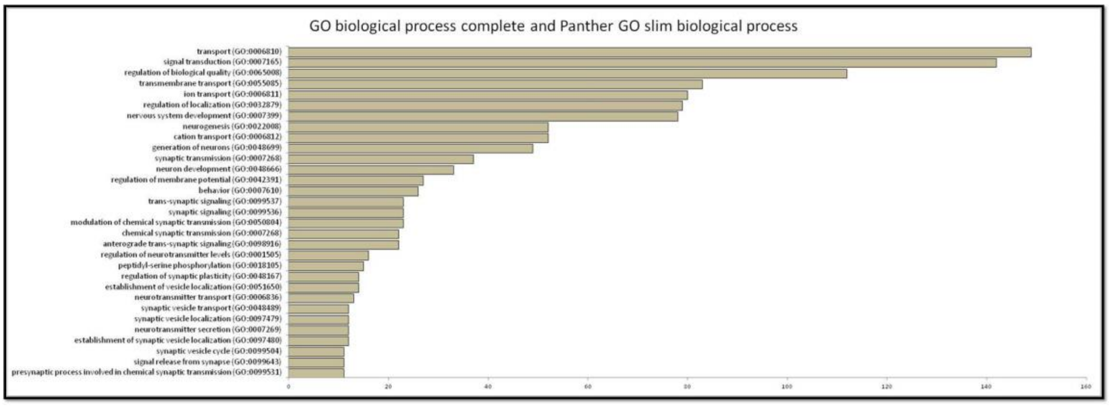

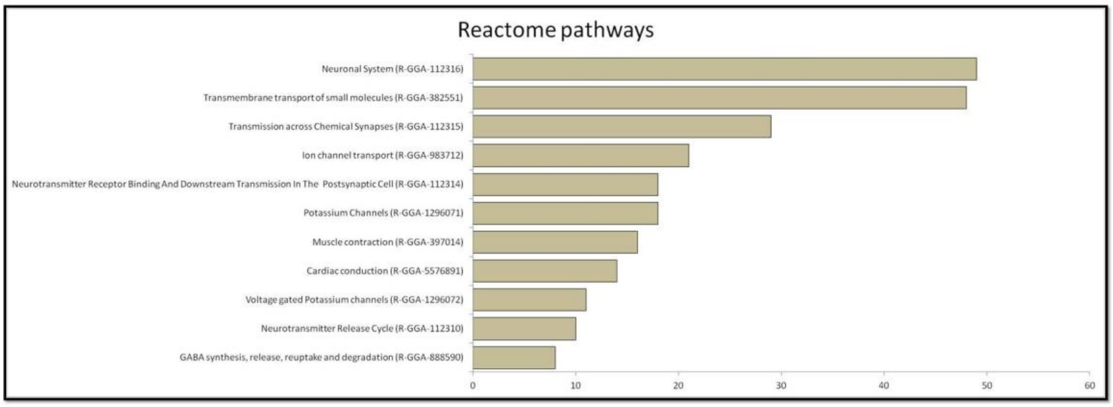

Figure S2: Reactome pathways and GO biological process generated by Panther enriched by genes showed higher expression level in 6-week-old cockerels.

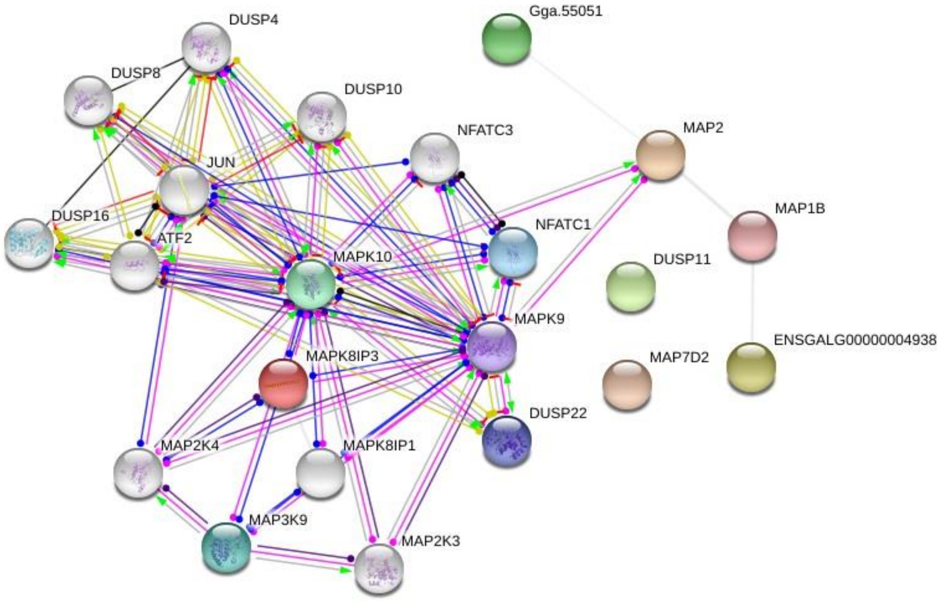

Figure S3: The connections of mitogen-activated protein kinase 10 (MAPK10). Colored genes were upregulated in 6-week-old cockerels and gray genes are background.
